# Computational analysis of high-risk SNPs in human *CHK2* gene responsible for hereditary breast cancer: A functional and structural impact

**DOI:** 10.1371/journal.pone.0220711

**Published:** 2019-08-09

**Authors:** Nutan V. Badgujar, Bhoomi V. Tarapara, Franky D. Shah

**Affiliations:** Stem Cell Biology Lab, Department of Cancer Biology, The Gujarat Cancer & Research Institute, Ahmedabad, Gujarat, India; Ohio State University Wexner Medical Center, UNITED STATES

## Abstract

Nowadays *CHK2* mutation is studied frequently in hereditary breast and ovarian cancer patients in addition to BRCA1/BRCA2. *CHK2* is a tumor suppressor gene that encodes a serine/threonine kinase, also involved in pathways such as DNA repair, cell cycle regulation and apoptosis in response to DNA damage. CHK2 is a well-studied moderate penetrance gene that correlates with third high risk susceptibility gene with an increased risk for breast cancer. Hence before planning large population study, it is better to scrutinize putative functional SNPs of *CHK2* using different computational tools. In this study, we have used various computational approaches to identify nsSNPs which are deleterious to the structure and/or function of CHK2 protein that might be causing this disease. Computational analysis was performed by different *in silico* tools including SIFT, Align GVGD, SNAP-2, PROVEAN, Poly-Phen-2, PANTHER, PhD-SNP, MUpro, iPTREE-STAB, Consurf, InterPro, NCBI Conserved Domain Search tool, ModPred, SPARKS-X, RAMPAGE, Verify-3D, FT Site, COACH and PyMol. Out of 78 nsSNP of human *CHK2* gene, seven nsSNPs were predicted functionally most significant SNPs. Among these seven nsSNP, p.Arg160Gly, p.Gly210Arg and p.Ser415Phe are highly conserved residues with conservation score of 9 and three nsSNP were predicted to be involved in post translational modification. The p.Arg160Gly and p.Gly210Arg may interfere in phosphopeptide binding site on FHA conserved domain. The p.Ser415Phe may interfere in formation of activation loop of protein-kinase domain and might interfere in interactions of *CHK2* with ligand. The study concludes that mutation of serine to phenylalanine at position 415 is a major mutation in native CHK2 protein which might contribute to its malfunction, ultimately causing disease. This is the first comprehensive study, where *CHK2* gene variants are analyzed using *in silico* tools hence it will be of great help while considering large scale studies and also in developing precision medicines related to these polymorphisms in the era of personalized medicine.

## Introduction

Of all cancers, one of the main cause of cancer related deaths is breast cancer among women worldwide, with 5% to 10% of cases being due to hereditary risk [1]. The *CHK2* gene is moderately penetrance gene most extensively studied as possible third high risk susceptibility gene in hereditary breast and ovarian cancer. *CHK2* gene is the human homolog of *Rad53* (*Saccharomyces cerevisiae*) and *Cds1* (*Schizosaccharomyces pombe*). Human *CHK2* gene is a tumor suppressor gene, located on long arm of chromosome 22 at q12.1 and encoded by CHK2 serine/threonine kinase. It consists of three major domains. 1) N-terminal has SQ/TQ cluster domain that serves as a site for phosphorylation in response to DNA damage, 2) forkhead-associated protein interaction domain (FHA) which is essential for activation in response to DNA damage and is rapidly phosphorylated in response to replication blocks and DNA damage. In FHA domain residues 112–175 are involved in dimerization of CHK2 molecules in phosphorylation manner, for full activation of CHK2 by trans-autophosphorylation of the activation loop. The major function of FHA domain is to regulate the kinase activities in CHK2 by interacting with other proteins thus mediates protein-protein interactions [2, 3] and 3) C-terminal which has serine/threonine kinase activity [4]. *CHK2* is activated by the kinases ATM and ATR in response to DNA double-strand breaks or replicative stress [5]. These proteins catalyze the phosphorylation of threonine 68 of CHK2 causing its transient dimerization via the FHA domain leading to *CHK2* trans-autophosphorylation and its full activation. In response to DNA damage, *CHK2* gene is involved in different pathways such as cell cycle regulation, DNA repair and apoptosis. *CHK2* phosphorylates downstream cell cycle regulators such as p53, Cdc25, and BRCA1 to activate checkpoint repair or recovery responses, as well as concurrently delay entry into mitosis [6, 7]. Deviation from its normal physiological function is likely to contribute to disease pathogenesis. In particular, the missense variants of *CHK2* p.Ile157Thr, p.Asp252Gly, c.1100delC, p.His371Tyr, p.Glu161del, p.Ser428Phe, c.591delA, p.Arg117Gly, p.Thr476Met and p.Asp438Tyr were significantly associated with germ-line variants in hereditary breast and ovarian cancer [8–11]. Finnish population (1.4%) and Polish population (0.2%) confer a relative risk for developing breast tumors of about 2 for women and 10 for men if c.1100delC mutation is present [12, 13]. Variant p.Ile157Thr, present in 5.3% of the Finnish population and in 4.8% of the Polish population, confers a relative risk of breast cancer of 1.5 [14, 15]. A recent analysis by the Breast Cancer Association Consortium (BCAC) estimated a relative risk of 2.26 for p.Thr367MetfsTer15 (rs555607708) [16]. Limited data is available for whole *CHK2* gene for hereditary breast and ovarian cancer. Further, impact of missense variants on protein function is not known fully, although substitutions in the FHA domain and the kinase domain have been shown to abolish activity [17–19].

Single nucleotide polymorphism is a common genetic variant in human and about 93% SNPs are present in human genes [20]. SNPs can be present in coding, noncoding or intergenic regions [21, 22]. Both non-coding and intergenic SNPs may have slight impact, but non-synonymous coding SNPs (nsSNPs) have more impact on protein [20]. Identification of the impact of variants on structure, stability and function of the protein is an important task as not all reported polymorphisms are deleterious [23]. Therefore there is a need to understand the deleterious impact of nsSNPs on protein structure and function using different recent molecular biology techniques. Till now large numbers of SNPs are reported in NCBI data, to screen these nsSNPS for their impact on biological function through experimental work is very tedious and costly. However, utilization of computational methods could be an efficient alternative for the same.

Nowadays, different computational tools have been extensively used for predicting deleterious nsSNP and their role in protein function, stability and structure maintenance. Taking all these in consideration, the present study is aimed to determine various deleterious nsSNPs of human *CHK2* gene using SIFT, Align GVGD, SNAP-2, PROVEAN, PolyPhen-2, PANTHER, PhD-SNP, I-Mutant, iPTREE-STAB, Mupro etc. Conservation of amino acid residues was predicted using ConSurf. ModPred was used to identify post-translational modification site present in protein. The 3D structure of the CHK2 protein was generated using SPARK-X and refined using ModRefiner. The quality of model was checked using RAMPAGE and Varify3D. The ligand binding sites were predicted using FTsite and COACH. The visualization of 3-D structure and labelling of native as well mutant amino acid was done using Pymol and Swiss PDB viewer.

## Materials and methods

### SNP dataset

The data related to human *CHK2* gene was retrieved from following databases: Uniport database (https://www.uniprot.org) (UniprotKB ID 096017), the NCBI database SNP (rsIDs) and FASTA nucleotide sequence (NC_000022.11) and amino acid sequence (NP_001005735) sequence from (https://www.ncbi.nlm.nih.gov) for further computational analysis [24, 25].

### Prediction of functional consequence of non-synonymous SNPs

The functional consequences of the nsSNP of human *CHK2* gene were analysed using different computational tools.

#### SIFT

SIFT (Sorting intolerant from tolerant) predicts whether an amino acid substitution affects protein function based on sequence homology and the physical properties of amino acids. SIFT can be applied to naturally occurring nonsynonymous polymorphisms and laboratory-induced missense variants. SIFT (http://siftdna.org/www/SIFT_dbSNP.html) determines if an amino acid substitution is deleterious to protein function [26]. A SIFT score predicts whether an amino acid substitution affects protein function. The SIFT score ranges from 0.0 (deleterious) to 1.0 (tolerated). The input query for SIFT algorithm is rsIds of SNPs from dbSNP.

#### Align GVGD

Align GVGD is a web based program available at http://agvgd.hci.utah.edu/. It combines the biophysical characteristics of amino acids, protein multiple sequence alignments to predict whether the missense substitution is deleterious or not [27]. The input query is FASTA sequence of protein and amino acid substitution.

#### SNAP2

SNAP2 (Screening of non-acceptable Polymorphism 2) predicts the functional consequences of amino acid variation based on neutral network classification method [28]. It is a web based tool available at https://www.rostlab.org/services/SNAP/ in which the input query is a protein sequence of CHK2 in FASTA format.

#### PROVEAN

PROVEAN (Protein variation effect analyzer) predicts whether single nucleotide variant affects protein function through alignment based score [29]. It is an online software available at http://provean.jcvi.org/index.php produced by J Craig Venture Institute. Based on this, if the score is below threshold value of 2.5, variant is predicted deleterious whereas the variant is neutral if the score is above 2.5. The input query is the FASTA sequence of protein CHK2 and amino acid variants.

#### PolyPhen-2

PolyPhen-2 (Polymorphism Phenotyping V2) predicts the impact of amino acid substitution on protein structure and function by using straight forward physical and comparative consideration [30]. It is a web based tool available online at http://genetics.bwh.harvard.edu/pph2/. It calculates the PSIC (Position-Specific independent score). If score is >0.85, then variant is probably damaging and score is >0.15 possibly damaging and rest are considered as benign. The input query for PolyPhen-2 is FASTA sequence of protein CHK2 and amino acid variants.

#### PANTHER

PANTHER cSNP (Protein analysis through evolutionary relationship- coding SNP) predicts functional consequences of variants on the protein. It is an online tool available at http://pantherdb.org/tools/csnpScoreForm.jsp. It compares the sequence of protein with a family of evolutionarily related protein. Longer the preservation time, higher the functional impact of amino acid variant. It calculates the subPSEC (Substitution Position Specific evolutionary conservation) score on the basis of alignment of evolutionary related proteins [31]. The input query is plain protein sequence, amino acid variants and human organism.

#### PhD-SNP

PhD-SNP (Predictor of human deleterious single nucleotide polymorphism) server is a Support Vector Machine (SVM) based method to discriminate between neutral and disease-related single point protein variants [32]. It is an online tool available at http://snps.biofold.org/phd-snp/phd-snp.html. Results were obtained through evolutionary information and using hybrid predictive model. The input query is plain protein sequence, position of SNP along with new residue.

#### MUpro

MUpro is a set of machine learning programs which predicts the protein stability changes for single nucleotide variation in amino acid sequence [33]. It is a web based server available at http://mupro.proteomics.ics.uci.edu/. Prediction of result based on both value and sign of energy change using SVM and sequence information only. The input query for this is also a plain sequence of protein followed by original and substituted amino acid.

#### iPTREE-STAB

iPTREE-STAB is a web based server available at http://203.64.84.190:8080/IPTREEr/iptree.html which is based on decision tree. It predicts the impact of single amino acid change on protein stability [34]. The input query is original amino acid as well as mutated amino acid residue followed by three flanking residues from both sides of the mutated residue.

### Phylogenetic conservation

Consurf is a computational tool available at http://consurf.tau.ac.il which calculates the evolutionary conservation of amino acid position through phylogenic relations between homologous sequences [35]. Consurf calculates conservation score from 0 to 9 which is classified into variable, average and highly conserved. The input query for consurf is FASTA sequence of protein CHK2.

### Prediction of post translational modification sites

The ModPred server is available at http://www.modpred.org which is used to predict post translational modification sites within CHK2 protein sequence. ModPred is a sequence-based predictor of potential post-translational modification (PTM) sites in proteins. It consists of 34 ensembles of logistic regression models, trained separately on a combined set of 126,036 non-redundant experimentally verified sites for 23 different modifications, obtained from public databases and an ad-hoc literature search [36].

### ExAC browser beta

ExAC browser is freely available at http://exac.broadinstitute.org. The minor allele frequency (MAF) was retrieved from ExAC Browser Beta for the nsSNPs of human *CHK2* gene. The Exome Aggregation Consortium (ExAC) is a coalition of investigators seeking to aggregate and harmonize exome sequencing data from a variety of large-scale sequencing projects and to make summary data available for the wider scientific community. The ExAC browser provides gene and transcriptcentric displays of variation, a critical view for clinical applications. Additionally, it provides a variant display, which includes population frequency and functional annotation data as well as short read support for the called variant. ExAC has already been used extensively by clinical laboratories worldwide [37]. The input query is name of human *CHK2* gene.

### Prediction of nsSNPs position in different protein domains

NCBI Conserved Domain Search tool (https://www.ncbi.nlm.nih.gov/Structure/cdd/wrpsb.cgi and InterPro (https://www.ebi.ac.uk/interpro/) were used to locate the position of SNPs in different domains of CHK2 protein structure [38, 39]. Input query for InterPro is a plain sequence of CHK2 and for NCBI Conserved Domain Search tool the input query is FASTA amino acid sequence of protein CHK2.

### Protein 3D modelling and structural analysis

The 3D structure of full length CHK2 protein is not available in protein data bank. The 3D structure of protein CHK2 was generated using SPARKS-X fold recognition server (http://sparks-lab.org/yueyang/server/SPARKS-X) [40]. The input query for SPARKS-X server is FASTA amino acid sequence of protein CHK2. The degree of similarity of templates used by SPARKS-X server for 3D model prediction was checked by BLASTp. The 3D structure predicted by SPARKS-X server was further refined using Modrefiner (https://zhanglab.ccmb.med.umich.edu/ModRefiner) [41]. The quality of refined model was checked using Varify3D (http://servicesn.mbi.ucla.edu/Verify3D) and RAMPAGE (http://mordred.bioc.cam.ac.uk/~rapper/rampage.php) [42]. Input query for Varify3D and RAMPAGE analysis is refined structure predicted using SPARKS-X.

### Ligand binding site prediction

The ligand binding sites within CHK2 protein were predicted using FT site server (http://ftsite.bu.edu/) and COACH server (https://zhanglab.ccmb.med.umich.edu/COACH/). FT site is freely available online tool which predicts ligand binding sites of CHK2 protein. FT site accurately identifies binding sites in over 94% of apoproteins, including structure based prediction of protein, the explanation of functional relationships among proteins, protein engineering and drug designing [43]. COACH is a meta-server based approach used for protein-ligand binding site prediction. Using two comparative methods, TM-SITE and S-SITE COACH predicts complementary ligand binding sites [44]. The input query for COACH is refined structure generated by modrefiner. PyMol and Swiss PDB viewer were used to visualize 3D structure of protein.

## Results

### SNP database

**Fig 1:**
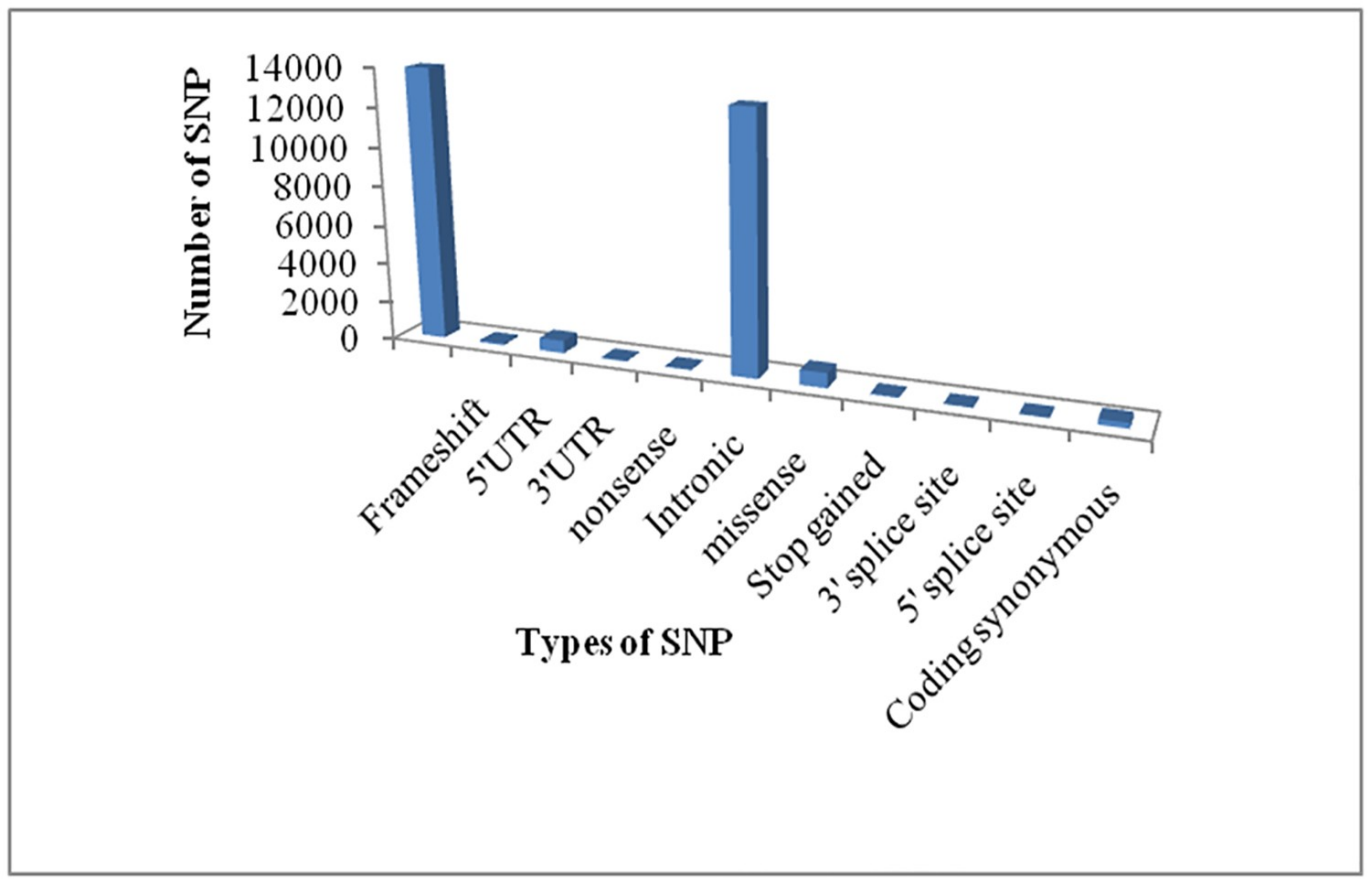
Distribution of SNPs in different functional classes of *CHK2* gene according to the dbSNP database.

The *CHK2* gene investigated in the present study was retrieved from dbSNP database (dbSNP- NCBI: https://www.ncbi.nlm.nih.gov/snp/?term=chek2). It contained a total of 13929 SNPs out of which 753 are missense (nsSNP), 105 are frame shift, 642 in 5'UTR, 55 in 3' UTR, 50 nonsense, 13062 intronic, 50 stop gained, 19 in 3' splice site, 24 in 5' splice site and 266 in coding synonymous SNPs (Fig 1). Only nsSNP of *CHK2* were selected for this investigation.

### Prediction of functional nsSNPs in *CHK2*

The *CHK2* single nucleotide variants obtained from dbSNP analysis were subjected to computational analysis through variety of tools. According to SIFT result out of 753 nsSNPs of *CHK2* gene total 78 SNP were predicted to be tolerated or deleterious and rest of 675 were not found in SIFT results. From these 78 SNPs, SIFT classified 35 nsSNPs as damaging, 43 as tolerated. To increase the accuracy of computational techniques, all the 78 SNPs predicted in SIFT were further validated by Align GVGD, SNAP2, PROVEAN, PolyPhen2 and PANTHER tools. Align GVGD is a method that combines Grantham Variation (GV) and Grantham Deviation (GD) scores to predicts whether the missense substitution is deleterious or not. In Align GVGD, if GD score is less than C15 then substitution is less likely affected and score is greater C65 then substitution is most likely affected. Out of 78 nsSNP Align GVGD predicted 43 SNPs as most likely affected and 10 nsSNPs as less likely affected. SNAP2 predicts whether the impact of amino acid variation is neutral or has effect on a query protein function by evaluating mutability landscape of the entire query protein sequence. Out of 78 SNPs subjected to SNAP2 prediction, 41 showed effect on protein function and 37 predicted as neutral SNPs. Among 78 SNPs subjected to PROVEAN analysis, 35 SNPs were predicted as deleterious and 43 SNPs were predicted as neutral. Out of 78 SNPs subjected to PolyPhen2 analysis 41 were predicted probably damaging, 10 predicted possibly damaging, 26 predicted benign and 1 was not predicted by PolyPhen2. For every input variant PolyPhen2 calculates PSIC (Position specific independent score). Out of 78 nsSNPs, 37 SNPs were predicted probably damaging, 17 predicted possibly damaging and rest 24 SNPs predicted probably benign by PANTHER cSNP. The nsSNP predicted as probably damaging by PolyPhen and PANTHER were considered as damaging and used for further analysis.

All the 78 nsSNPs of *CHK2* gene were further analyzed for correlation with disease after functional impact through PhD-SNP. PhD-SNP is a SVM based classifier which predicts the result through evolutionary information and hybrid predictive method with the accuracy of 78% of human protein [29]. PhD-SNP revealed the most unique results showing only 20 nsSNPs as diseased and rest of 58 SNPs as neutral.

We predicted any stability alteration in the CHK2 protein with the help of MuPro and iPTREE-STAB which predict the result by considering single site variant. MuPro predicted 56 nsSNP which decrease stability of CHK2 protein and rest of 22 SNPs increase stability. iPTREE-STAB result revealed to decrease stability of 74 nsSNP and 4 nsSNP showed increase in protein stability. According to some studies, decreased protein stability causes increase in degradation, misfolding and aggregation of proteins. We shortlisted those nsSNP which are common in all 9 different algorithm tools and predicted as deleterious SNPs. Total 7 SNPs out of 78 SNPs met the criteria and classified them as high risk and selected for further analysis. Result of SIFT, Align GVGD, SNAP2, PROVEAN, PANTHER, Ph-D SNP, MuPro and iPTREE-STAB is shown in Table 1.

**Table 1:** Prediction of functional consequences of nsSNP in human *CHK2*.

SNPs rs ID	AA Variant	SIFT	Align GVGD	SNAP2	PRO VEAN	Poly Phen-2	Panther	Ph-D SNP	MuPro	iPTREE-STAB
rs17879961	I200T	T	C65	Effect	N	PosD	PosD	N-1	↓	-ve
rs17882942	L555V	T	C25	Neutral	N	Ben.	ProB	N-9	↓	-ve
rs17883172	E544K	T	C55	Neutral	N	Ben.	ProB	N-3	↓	-ve
rs17883862	P85L	T	C65	Effect	N	ProD	PosD	N-6	↑	-ve
rs17886163	I491S	T	C65	Neutral	N	Ben.	PosD	N-5	↓	-ve
rs28909980	D390N	D	C15	Effect	D	ProD	ProD	N-0	↓	-ve
rs28909982	R160G	D	C65	Effect	D	ProD	ProD	Di-5	↓	-ve
rs72552322	G210R	D	C65	Effect	D	ProD	ProD	Di-7	↓	-ve
rs72552323	I203T	D	C65	Effect	D	ProD	ProD	Di-2	↓	-ve
rs77130927	R223C	D	C65	Neutral	D	PosD	PosD	Di-10	↓	-ve
rs121908694	S41F	D	C65	Effect	N	ProD	ProD	N-6	↓	-ve
rs121908701	R224H	T	C25	Neutral	N	Ben.	ProB	Di-2	↓	-ve
rs121908702	E282K	T	C55	Neutral	N	ProD	PosD	Di-2	↓	-ve
rs121908703	S399L	T	C65	Neutral	D	PosD	PosD	N-2	↑	-ve
rs121908704	T444A	T	C55	Neutral	N	Ben.	ProB	N-6	↓	-ve
rs121908705	N489D	T	C15	Neutral	N	Ben.	ProB	N-7	↓	-ve
rs121908706	R517H	T	C25	Effect	D	ProD	ProD	Di-7	↓	-ve
rs137853007	R188W	D	C65	Effect	D	ProD	ProD	Di-2	↓	-ve
rs137853008	A17S	T	C65	Neutral	N	Ben	ProB	N-5	↓	-ve
rs137853009	R223H	D	C25	Neutral	D	Ben	PosD	Di-4	↓	-ve
rs137853010	R224C	T	C65	Neutral	N	Ben	ProB	Di-4	↓	-ve
rs137853011	S471F	T	C65	Neutral	D	ProD	PosD	Di-1	↑	-ve
rs138040612	E571K	T	C55	Neutral	N	ProD	PosD	N-6	↓	-ve
rs139088611	V494A	T	C55	Neutral	N	Ben	ProB	Di-0	↓	-ve
rs139366548	Y467H	T	C65	Effect	D	ProD	ProD	N-8	↓	-ve
rs141568342	E64K	D	C55	Effect	N	Ben	ProB	N-6	↓	-ve
rs141776984	C286R	T	C65	Effect	D	PosD	ProD	Di-1	↓	-ve
rs142243299	V25I	T	C25	Neutral	N	Ben	ProB	N-5	↓	-ve
rs142763740	T519M	D	C65	Effect	D	ProD	PosD	N-7	↓	-ve
rs143611747	R361H	T	C25	Neutral	N	Ben	PosD	N-6	↓	-ve
rs143965148	D540N	T	C15	Neutral	N	Ben	ProB	N-8	↓	-ve
rs144850845	G210E	D	C65	Effect	D	ProD	ProD	N-1	↓	-ve
rs145324174	C428Y	D	C65	Effect	D	ProD	ProD	N-1	↓	-ve
rs146198085	N229H	D	C65	Neutral	D	ProD	ProD	Di-8	↓	-ve
rs147877722	S415F	D	C65	Effect	D	ProD	ProD	Di-5	↓	-ve
rs148053495	R361C	D	C65	Effect	D	ProD	PosD	Di-3	↓	-ve
rs149501505	R566C	T	C65	Effect	N	ProD	ProB	N-5	↓	-ve
rs149991239	T59K	D	C65	Effect	D	ProD	ProD	N-4	↓	-ve
rs199708878	R3W	D	C65	Effect	N	ProD	ProD	N-8	↓	-ve
rs199749372	I264V	T	C25	Neutral	N	Ben	ProD	N-8	↓	-ve
rs199859140	D404H	T	C65	Neutral	N	PosD	ProB	Di-7	↓	-ve
rs200050883	D481Y	D	C65	Effect	D	PosD	PosD	Di-7	↓	-ve
rs200432447	R562G	D	C65	Effect	D	Ben	ProD	N-2	↓	-ve
rs200451612	I264M	T	C0	Neutral	N	PosD	ProD	N-3	↓	-ve
rs200649225	R449H	T	C25	Neutral	N	ProD	PosD	N-3	↓	-ve
rs200928781	Y433C	D	C65	Effect	D	ProD	ProD	N-6	↓	-ve
rs201084748	S5L	T	C65	Effect	N	Ben	ProB	N-8	↓	-ve
rs201206424	R389C	D	C65	Effect	D	ProD	ProD	N-7	↓	-ve
rs202051128	I387M	T	C0	Effect	N	ProD	ProD	N-7	↓	-ve
rs202089930	T426I	D	C65	Effect	D	ProD	ProD	N-1	↓	-ve
rs267606211	S422F	D	C65	Effect	D	PosD	ProD	N-2	↑	+ve
rs368570187	R180Q	T	C35	Neutral	N	Ben	ProB	N-7	↓	-ve
rs369070738	N448S	T	C45	Neutral	N	Ben	ProB	N-2	↓	-ve
rs369223840	N229S	T	C45	Neutral	D	ProD	ProD	N-4	↓	-ve
rs369256181	Q11R	T	C35	Neutral	N	Ben	ProD	N-7	↓	-ve
rs370968992	F518I	T	C15	Neutral	N	Ben	ProB	N-7	↓	-ve
rs371207635	H382Y	D	C65	Effect	D	ProD	ProD	N-6	↓	-ve
rs371657037	S53T	D	C55	Neutral	N	ProD	ProD	N-5	↓	-ve
rs372874441	D177H	T	C65	Neutral	N	ProD	ProB	N-8	↓	-ve
rs373073383	A435V	D	C55	Effect	D	ProD	ProD	N-8	↓	-ve
rs373648967	K162R	T	C25	Neutral	N	Ben	ProB	N-6	↓	+ve
rs373959274	R564Q	D	C35	Effect	N	ProD	ProD	N-3	↓	-ve
rs374395284	E364A	D	C65	Effect	D	ProD	ProD	N-4	↓	-ve
rs374660293	L381H	D	C65	Effect	D	ProD	ProD	N-4	↓	-ve
rs375130261	M424V	D	C15	Effect	D	ProD	ProD	N-8	↓	-ve
rs17880867	N489K	T	C65	Neutral	N	Ben	ProB	N-4	↓	-ve
rs17881473	F490I	D	C15	Effect	D	Ben	ProD	N-8	↓	-ve
rs17882922	L479M	D	C0	Neutral	N	ProD	ProD	N-1	↓	-ve
rs112032663	G30D	T	C65	Effect	N	ProD	ProB	N-3	↓	+ve
rs113947614	I264T	T	C65	Neutral	N	Ben	ProD	N-4	↓	-ve
rs137926355	R144Q	T	C35	Neutral	N	-	-	N-8	↓	-ve
rs141502354	I386V	T	C25	Neutral	N	PosD	PosD	N-6	↓	-ve
rs142966756	R191M	D	C65	Effect	D	ProD	PosD	N-5	↓	-ve
rs150677496	L173Q	T	C65	Effect	D	ProD	PosD	Di-5	↓	-ve
rs151218932	C18Y	T	C65	Effect	N	Ben	ProB	N-4	↓	-ve
rs372168051	P225H	D	C65	Effect	D	ProD	ProD	Di-5	↓	-ve
rs375507194	Q20H	D	C15	Neutral	N	ProD	ProB	N-6	↓	-ve
rs376736188	Q27E	T	C25	Effect	N	PosD	ProB	N-9	↓	+ve

Where **D**: Deleterious; **T**: Tolerated; GD ≥C65 = most likely affected; GD ≥C0 = less likely affected; **N**: Neutral; **D**: Deleterious; **ProD**: Probably damaging; **ProB**: Probably benign; **PosD**: Possibly damaging; **Ben**: Benign; **N**: Neutral; **Di**: Disease: **↓**: Decrease; **↑**: Increase; -**ve**: negative; **+ve**: positive

### Conservation profile of deleterious nsSNP in *CHK2*

**Fig 2:**
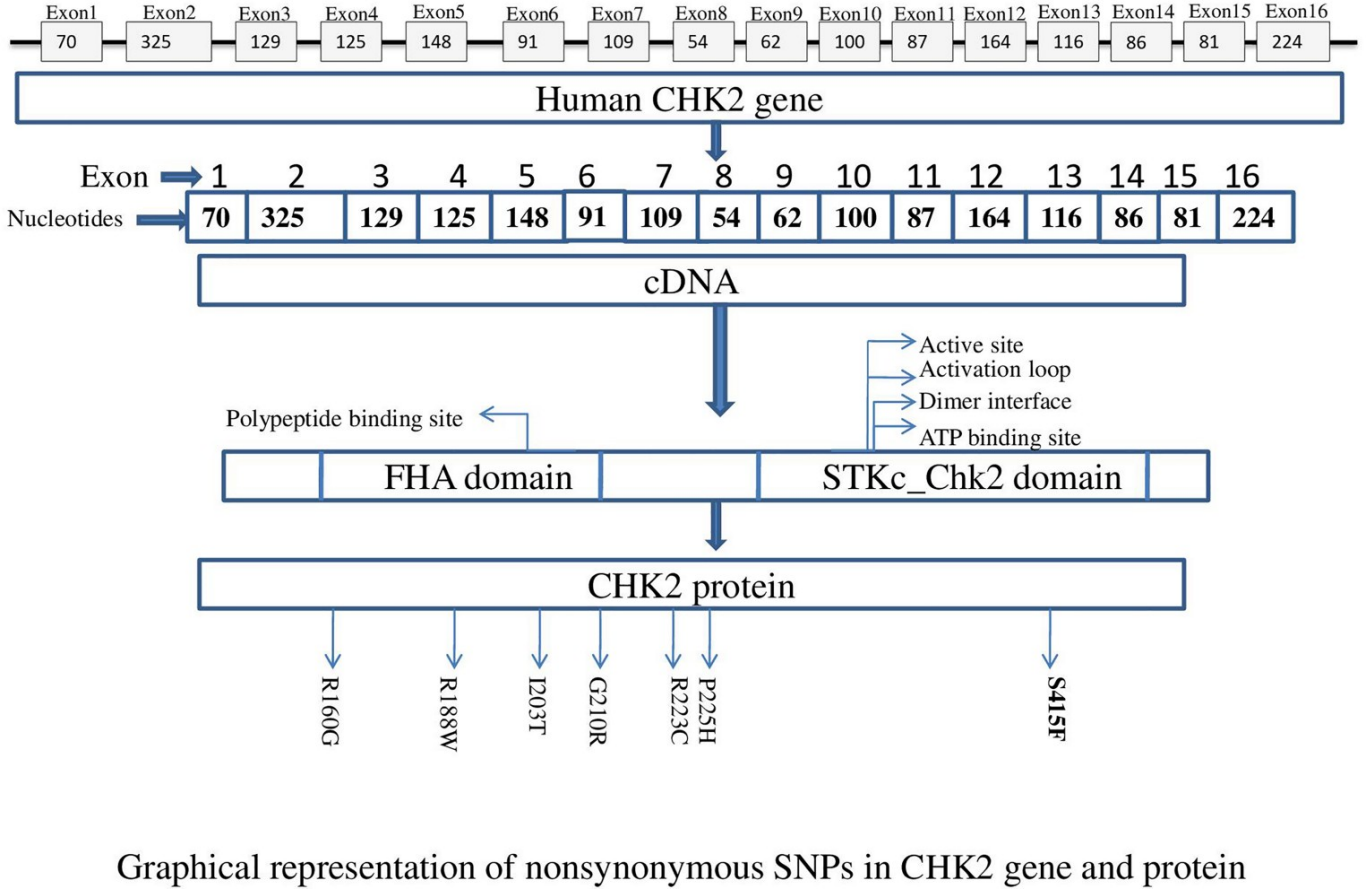
Graphical representation of the position of nsSNP in *CHK2* gene and protein.

Evolutionary information is used to predict whether the substitution of amino acid affects the protein functions or not. Consurf web server was used to calculate the conservation score of amino acid residue of CHK2 protein to further analyze possible effect of 7 most deleterious nsSNP predicted through different computational tool. Results were obtained in the form of structural representation of the protein (S1 Fig). Highly conserved residues are predicted as either functional or structural based on their location either on protein surface or inside its core. Results obtained via conSurf represented all residues of CHK2 showing their structural and functional conservation levels. But we focused only on those residues which matched their positions with 7 high risk nsSNPs which we have identified. Taking this into consideration, those nsSNPs which are located at these conserved regions are considered immensely damaging to protein as compared to those at non-conserved sites [45, 46]. According to consurf output, p.Arg160Gly, p.Gly210Arg, p.Ser415Phe are highly conserved residues with conservation score of 9. Four amino acids were predicted average conserved. The result of consurf is shown in Table 2. The summary of deleterious prediction for each SNP is shown in Fig 2.

**Table 2:** ConSurf predictions of most deleterious nsSNP showing conservation profile and their post translation sites prediction by ModPred and their clinical significance in clinvar with their minor allelic frequency (MAF).

SNP ID	Residue and Position	Conser-vation score	B/E	F/S	PTM	Clinvar	MAF
rs28909982	R160G	9	E	F	Proteolytic cleavage	Conflicting-interpretations-of-pathogenicity, likely-pathogenic	0.0001320
rs137853007	R188W	6	E	F	Proteolytic cleavage	not-provided, pathogenic, likely-benign, likely-pathogenic	0.00003296
rs72552323	I203T	6	B	-	-	Variable of uncertain-significance	0.000008240
rs72552322	G210R	9	E	-	-	uncertain-significance, conflicting-interpretations of pathogenicity, likely-pathogenic	0.00004120
rs77130927	R223C	5	E	-	ADP ribosylation	uncertain-significance, conflicting-interpretations of pathogenicity	0.001360
rs372168051	P225H	5	B	S	-	uncertain-significance	-
rs147877722	S415F	9	E	-	-	uncertain-significance	-

**B**: Buried; **E**: exposed; **F**: functional; **S**: structural; **PTM**: post translation modification site; **MAF**: minor allele frequency

### Prediction of post translational modification sites

Post translational modification sites present within human CHK2 protein were predicted using ModPred. Out of 7 most significant nsSNPs, three amino acids p.Arg160Gly, p.Arg223Cys, p.Arg188Trp were predicted to be involved in post translational modification sites including proteolytic cleavage and ADP ribosylation. The results of modpred are shown in Table 2.

### ExAC

The minor allele frequency (MAF) was retrieved from ExAC Browser Beta (http://exac.broadinstitute.org/gene/ENSG00000183765) for the nsSNPs of human *CHK2* gene. The result of minor allele frequency of nsSNPs is shown in Table 2.

### Prediction of nsSNPs position in different protein domains

According to Interpro and NCBI Conserved Domain Search tool two major domains were predicted in CHK2 protein. One was STKc_Chk2 domain (serine/threonine kinase, cell cycle checkpoint kinase 2) which comprises 256–529 amino acids and another one was FHA domain (Forkhead associated domain) which comprises 156 to 244 amino acids. In CHK2 amino acid sequences 269–411 were predicted catalytic domain of ATP Binding site; 264–471 were predicted catalytic domain of dimer interface; 269–434 amino acid sequences were present in active site; 273–434 amino acid sequences were present in polypeptide substrate binding site. The 22 amino acid residues present in activation loop (Thr, Asp, Phe, Gly, His, **Ser at 415**, Lys, Ile, Leu, Gly, Glu, Thr, Ser, Leu, Met, Arg, Thr, Leu, Cys, Gly, Thr, Pro, Thr) of STKc_Chk2 domain. The 160 to 210 amino acid sequences present in polypeptide binding site on conserved domain of FHA domain (**Arg at 160**^**th**^ and **Gly at 210**^**th**^ position).

### Protein 3D modeling and structural analysis

The 3D structure of full length CHK2 protein was not available in protein data bank. SPARKS-X modeled 3D structure of CHK2 protein by submitting FASTA amino acid sequences, where 10 best full length models were generated using different similar templates. The quality of full length models were predicted based on (>6) Z-score. All the templates were subjected to BLASTp analysis to identify the sequence similarity of the templates with CHK2 protein. The 3D structure generated using 3i6wA as a template was used for further analysis. The 3D structure was further refined by submitting structure in ModRefiner server which showed RMSD value 2.821 and TM-score of 0.9685 to initial model. After that refined structure was further validated using Verify3D and RAMPAGE tools. Ramachandran plot analysis by RAMAPAGE for the native protein model showed 541 (92.6%) residues in favoured region, 38 (6.5%) residues in allowed region and 5 (0.9) residues in outlier region. Varify-3D showed 66.21% of the amino acids have scored ≥0.2 in 3D-1D profile. The results of both tools are shown in Table 3.

**Table 3:** Validation of protein structure.

Varify3D	RAMPAGE		
Percentage of the amino acids have scored ≥0.2 in 3D-1D profile	Favoured region	Allowed region	Outlier region
66.21%	541 (92.6%)	38 (6.5%)	5 (0.9)

### Ligand binding site prediction

**Fig 3:**
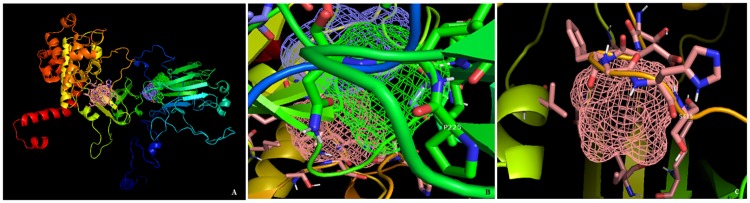
Ft site prediction showing Ser at 415 and Pro at 225 positions in 1^st^ and 2^nd^ ligand binding site respectively. A) Pink, green and purple coloured mesh are 1^st^, 2^nd^ and 3^rd^ ligand binding site respectively of human CHK2 protein predicted using FT site server B) Zoom in on interaction at Pro 225 C) Zoom in on at Ser 415.

FT site server predicted 3 binding sites present in CHK2 protein. First binding site consisted residues Lys at 292^th^, Leu at 320^th^, Ile at 329^th^, Ile at 342^th^, Ile at 331^th^, Leu at 344^th^, Thr at 410^th^, Asp at 411^th^, Phe at 412^th^, Gly at 413^th^,His at 414 and **Ser at 415**^**th**^ position. Second binding site consisted residues Trp at 93^th^, Tyr at 199^th^, Ile at 200^th^, Ala at 201^th^, **Pro at 225**^**th**^, Leu at 226^th^, Asn at 227^th^ and Asp at 246^th^ position. Third binding site constituted Trp at 93^th^, Asn at 197^th^, Ser at 198^th^, Tyr at 199^th^, Asp at 246^th^, Thr at 248^th^ and Val at 249^th^ position. Two binding sites are presented using PyMOL in Fig 3. Coach server also predicted **Ser at 415** within ligand binding sites. The detailed results of COACH prediction are shown in Table 4.

**Table 4:** Prediction of ligand binding sites within CHK2 protein using COACH.

**COACH Result**			
C-Score	Cluster Size	Name of ligands	Residue number
0.88	2636	MP6	269,270,271,277,290,292,329,344,345,346,347,348,350,351,394,395,397,410,411
0.07	148	2K5	269,277,290,292,316,320,329,342,344,345,346,347,350,397,411,412
0.03	73	MG	272,392,395,411
0.01	58	07Q	269,272,275,276,277,290,292,293,294,344,345,346,347,411
0.01	52	PEPTIDE	271,273,351,353,357,361,390,392,393,394,**415**,430,431,432,462,466,467,468,471,472,473,479,480
0.01	24	1RA	270,271,272,277,290,292,316,320,329,342,344,345,346,410,411,412,416
0.0	1	MG	312,315,413
0.0	1	CA	535,536,537
0.0	2	CA	282,333
0.0	11	MG	292,316,411,**415**
**TM-site**			
C-Score	Cluster Size	Name of ligands	Residue number
0.58	308	ANP, ADP, ATP	269,270,271,277,290,292,329,344,345,346,347,348,350,351,394,395,397,410,411
0.29	11	AMP,FMM,I76	269,277,290,292,316,320,329,342,344,345,346,347,350,397,411,412
0.20	30	III	271,272,273,274,351,353,357,392,393,394,**415**,431,432,434,462,467,468,471,472,473,479,480
0.19	9	Mg, ANP, B11	292,395,411
0.18	5	AF3, MG, PO4	272,273,274,390,392,395,411
**S-Site**			
C-Score	Cluster Size	Name of ligands	Residue number
0.41	488	ANP, ADP, ATP	267,269,270,271,272,273,274,275,277,290,292,316,329,344,345,346,347,348,350,351,354,394,395,397,410,411
0.19	66	MG, MN, IMD	273,274,292,351,390,392,394,395,410,411
0.14	27	MG, 7PE, MN	269,270,271,273,274,275,277,290,292,294,304,309,312,313,316,317,320,329,342,344,345,346,347,397,410,411,412,413,414,**415**,416,423
0.13	18	PDY, IMD, AGX	353,354,355,356,357,358,359,393,394,433,462,466,467,468,469,470,471,472
0.11	9	III, TAR	351,353,354,356,357,360,390,392,393,394,411,414,427,428,429,430,431,432,434,438,439,440,441,462,466,467,468,469,470,471,476,477,479,480,482,488,489,491,492,493,494
**FINDSITE**			
C-Score	Cluster Size	Name of ligands	Residue number
0.82	115	Site 1	269,270,272,274,275,277,290,292,294,329,344,346,347,351,395,397,410,411
0.04	6	Site 2	269,270,272,273,274,275,277,290,292,305,312,329,344,346,347,351,353,357,390,392,393,394,397,410,411,**415**,427,428,429,430,431,432,433,434,462,468,471
0.04	5	Site 3	325,376,379,380,524,535,536,539,540
0.04	5	Site 4	273,302,303,305,351,353,354,357,390,392,393,394,415,427,428,429,430,431,432,433,434,462,467,468,471,473,476,479,480
0.01	2	Site 5	428,430,434,438,439,476,480

### 3D structure prediction of mutant and model validation

The 3D structure of mutant of CHK2 protein was generated by substituting serine with phenylalanine at 415^th^ position in wild type sequence and the sequence was submitted to SPARKS-X server. The 3D structure generated was further refined by submitting structure in ModRefiner server which showed RMSD value 2.310 and TM score of 0.9517. The prediction of TM score suggested the structural deviation of mutant protein as compared to native. After that refined structure was further validated using Verify3D and RAMPAGE. Verify 3D showed 60.75% of the amino acids have scored ≥0.2 in 3D-1D profile. Mutant model is a good quality as having more than 90% region in favoured region. Mutant model showed (93.3%) residues in favoured region, 31 residues (5.3%) residues in allowed region and 8 (1.4%) residues in outlier region.

## Discussion

The *CHK2* gene is a tumor suppressor gene, involved in cell-cycle regulation, in response to DNA damage, DNA repair and apoptosis pathway. Variants of *CHK2* have been implicated in various types of cancer including breast cancer [47]. Single nucleotide polymorphism plays an important role in most of the diseases. About more than 4 million unique human single nucleotide polymorphism (SNPs) have been described by dbSNPs and 2% of the reported SNPs associate with monogenic diseases are present in protein coding region and hence predicted that these SNPs can be related to complex inherited disease traits [48]. Testing the functional consequences of variant by using functional assay can be the best approach but it is quite costly and time consuming too. Hence, for this purpose we have exploited computational approach by using various *in silico* tools of different algorithms for the analysis of SNVs in *CHK2* gene. To date, 13929 human *CHK2* gene SNPs are reported in NCBI dbSNP (database) which have been located in non-coding, coding and regulatory regions. The coding SNVs cause amino acid variation which further alters the protein function and leads to disease susceptibility. All the nsSNPs may not have major deleterious effect on protein function, some may have neutral effect. Therefore it is necessary to differentiate deleterious SNPs from the neutral SNPs to analyze susceptibility of individual SNPs to diseases, and also to focus on those SNVs which are responsible for structural and functional consequences of CHK2 protein [49]. However, to predict the pathogenic effect of nsSNP using single bioinformatic tool may not be reliable [50]. In present study prediction of *CHK2* genetic variants was accomplished by utilizing sequence and structure based bioinformatics tools- SIFT, Align GVGD, SNAP2, PolyPhen 2, PROVEAN, PANTHER, PhD SNP, MuPro and iPTREE-STAB. According to study of Hicks et al., and Thusberg and Vihinen, to identify most deleterious nsSNPs, SIFT and PolyPhen 2 were reported as best performing tools [51, 52]. To check the stability of protein, MuPro and iPTREE-STAB were used. Out of 79 nsSNP subjected to functional analysis 7 SNPs (p.Arg160Gly, p.Arg188Trp, p.Ile203Thr, p.Gly210Arg, p.Arg223Cys, p.Pro225His and p.Ser415Phe) were predicted to be most deleterious nsSNP in human CHK2 protein. To the best of our knowledge none of the studies showed the genetic risk of p.Arg160Gly, p.Arg188Try, p.Ile203Thr, p.Gly210Arg, p.Arg223Cys, p.Pro225His and p.Ser415phe with any known disease condition. p.Arg160Gly, p.Arg188Try, p.Ile203Thr, p.Gly210Arg, p.Arg223Cys and p.Pro225His all nsSNP are part of FHA domain which is activated in response to DNA damage. p.Arg160Gly marks the substitution of arginine (basic amino acid) by glycine (non-polar amino acid) and vice-versa in p.Gly210Arg substitution. p.Ile203Thr entails the substitution of isoleucine (nonpolar) to threonine (-OH containing amino acids) leading to decrease in stability of protein. In FHA domain, 2 SNPs (p.Arg223Cysand p.Arg188Try) leads to substitution of arginine (basic amino acids) to cysteine (sulphar containing amino acid) and tryptophan (nonpolar aromatic amino acid) which decrease protein stability. R160, G210, S415 are highly conserved residues with conservation score of 9. Four amino acids (R160, I203, R223 and P225) were predicted average conserved. p.Arg160Gly, p.Gly210Arg and p.Arg223Cys might interfere in post-translational modification of CHK2 protein as these residues were predicted to be involved in post translational modifications through ModPred. The Arg at 160 and Gly at 210 residue present in polypeptide binding site on conserved site of FHA domain. Ser 415 residue is present in STKc_Chk2 domain. In p.Ser415Phe substitution of serine (-OH containing amino acid) to phenyl alanine (non-polar amino acid). This residue is part of aimer interface, catalytic domain of ATP binding site and active site of STKc_Chk2 domain. Any change in this residue alters the stability of protein which is predicted by Mupro and iPTREE_STAB. Two amino acids S415 and P225 were predicted to be involved in ligand binding site interactions. These suggest that p.Pro225His and p.Ser415Phe might interfere in ligand binding site interactions. Several studies have investigated the role of *CHK2* polymorphism as a genetic determinant for susceptibility to diseases. Several polymorphisms (p.Ile157Val, p.Asp252Gly, c.1100delC, p.Asp438Tyr and p.His371Tyr) have been reported for the *CHK2* gene [53, 54]. Pritzlaff and their colleagues assessed multi-gene panel testing using male breast cancer patients and identified pathogenic variants i.e.c.591delA, p.Arg117Gly, p.Thr476Met, p.Ser428Phe, p.Iso157Thr, p.Gln29* andc.1100delC in different population [55]. CHK2*c.1100delC and p.Ile157Thr were most studied in populations all over the world. The inherited variants *CHK2* c.1100delC truncates the kinase domain of the CHK2 protein and is responsible for a two fold increase in breast cancer risk in families of northern and north-western European ancestry [12, 13, 56]. According to Delimitsou and his colleagues study, p.Ile160Arg and p.Ile160Thr variants were characterized as damaging and p.Asp203Gly variant was characterized as benign. All these variants were located within the kinase domain [57]. p.Ile160Arg was characterized as intermediate according to study done by Roeb et al in 2012 [58]. Different *CHK2* variants were categorized as damaging according to *in silico* tools and yeast based assay i.e p.Trp93Arg, p.Cys108Arg, p.Arg117Gly, p.Arg145Trp, p.Arg148Gly, p.Ile160Arg, p.Ile160Thr, p.Asp162Gly, p.Asn166Ser, p.Gly167Arg, p.Leu183Ser, p.Leu183Phe, p.Leu236Pro, p.Ile251Phe, p.Arg346Cys, p.Arg346His, p.Asp347Ala, p.Asn352Asp, p.Gly370Glu, p.Cys385Arg, p.Thr387Ser, p.Tyr390Ser, p.Ala392Pro, p.Ala392Val, p.Glu394Lys, p.Cys420Thr, p.Tyr424His, p.Arg474Cys and p.His483Arg [57]. Avraham Shaag and his team discovered two novel amino acid substitutions, p.Ser428Phe in the kinase domain and p.Pro85Leu in the N-terminal region [59]. The individual having *CHK2* sequence variants (c.1100delC) may contribute to the Li-Fraumeni syndrome in Dutch families [60]. In Pakistan, two novel mutations p.Gln20X and p.Glu85X at exons 1 and 2 respectively have been identified in breast cancer patients [61]. However the results were contradictory among different studies. By means of *in silico*, deleterious prediction done in the present study, the p.Gln20His and p.Pro85Leu were not predicted highly deleterious. However, Pro at 85 and Gln at 20^th^ were predicted as conserved residue with conservation score of 7. However none of the study till now available that identify p.Pro225His and p.Ser415Phe nsSNPs as damaging. *CHK2* variants found in this study have not been reported earlier so they need to be validated to check its significance. The major limitation of this study is the fact that it is *in silico* study thus the results cannot be blindly extrapolated to humans without validation by wet lab study. When we predict pathogenicity of *CHK2* variants, it is important to conduct functional assay in cell-lines. In addition to this, analysing data from epidemiological and genetic studies as well as segregation analysis would provide more accurate classification.

As multiple *CHK2* variants of unknown clinical significance emerge every day when performing genetic testing analyses in patients with cancer, a rapid variant assessment is of great importance. Therefore, the *in silico* assay used herein provides essential, fast and low ‐cost evaluation for the largest series of tested *CHK2* variants to date, thus providing valuable information that can be ultimately implemented in clinical practice. Thus, the present study indicates that the procedure of computational approach provides an alternative approach to select SNPs targets by considering the role of SNPs on the functional attributes or molecular phenotype of protein. These results may be helpful for further understanding of *CHK2* SNPs in disease susceptibility by laboratory experiments.

## Conclusion

The present study suggests that structure and function of *CHK2* can be distributed by various nsSNPs. In native protein of *CHK2* gene, out of 79 SNPs, seven major variants found were: p.Arg160Gly, p.Arg188Trp, p.Ile203Thr, p.Gly210Arg, p.Arg223Cys, p.Pro225His and p.Ser415Phe. Among seven most significant SNPs, 3 were highly conserved and 4 SNPs were averaged conserved residues. Among 7 most significant SNPs, 3 were predicted to be involved in post translational modifications. A variant of Serine→Phenyl alanine at position 415 occurs in activation loop of protein-kinase domain of CHK2 protein hence is of particular concern as this is the functional domain of the protein. The one SNP p.Ser415Phe might interfere in interactions of CHK2 with ligand. Therefore, these nsSNPs can be strongly considered as key candidates in causing diseases related to *CHK2* malfunction and hence will help in effective drug discovery and developing precision medicines. Wet lab experiments are needed to explore the effects of these polymorphisms on structure and function of protein.

## Supporting information

S1 Fig Consurf prediction showing conservation profile of amino acids in CHK2 gene. (PDF)

## Abbreviations

3′UTR: Three prime untranslated region
5′UTR: Five prime untranslated region
dbSNP: Database of SNP
MAF: Minor allele frequency
NCBI: National Centre for Biotechnology Information
nsSNPs: Non-synonymous SNPs
PDB: Protein Data Bank
PhD-SNP: Predictor of human deleterious single nucleotide polymorphisms
PolyPhen-2: Polymorphism phenotyping v2
PROVEAN: Protein Variation Effect Analyzer
SIFT: Sorting intolerant from tolerant
SNVs: Single nucleotide variations
SVM: Support vector machine
