# Supplementary material for: Computational analysis of high-risk SNPs in human CHK2 gene responsible for hereditary breast cancer: A functional and structural impact
Source: PLoS One. 2019 Aug 9;14(8):e0220711. doi: 10.1371/journal.pone.0220711 (PMC6688789; doi:10.1371/journal.pone.0220711)
Supplement: S1 Fig — (PDF) [file pone.0220711.s001.pdf]

ConSurf Results

```
1
MSRESDVEAQ
eeeeeeeebe

11
QSHGSSACSQ
eeeeeebbee

21
PHGSVTQSQG
eeeeeeeeee

31
SSSQSQGISS
eeeeeeebeb

41
SSTSTMPNSS
eeeeeeeeee

51
QSSHSSSGTL
eeeeebebeb

61
SSLETVSTQE
eebeebbeee

71
LYSIPEDQEP
bbbbeeeeee

81
EDQEPEEPTP
eeeeeeeeee

91
APWARLWALQ
ebbbbbbbbe

101
DGFANLETES
eebeebeeee

111
GHVTQSDLEL
eeeeeeeeee

121
LLSSDPPASA
eeeeeeeeee

131
SQSAGIRGVR
eeeeeeeebb

141
HHPRPVCSLK
eeeeebeebe

151
CVNDNYWFGR
beeeeeebee
        ff

161
DKSCEYCFDE
eeebebbbee

171
PLLKRTDKYR
eeeeeeeebe

181
TYSKKHFRIF
ebbeeebebe
  sf f

191
REVGPKNSYI
eeeeeeeeeb

201
AYIEDHSGNG
bbbeeeeeee
    f f ff

211
TFVNTELVGK
ebbeeeeeee
f  f    f

221
GKRRPLNNNS
eeeeebeeee
     s f f

231
EIALSLSRNK
ebbbbeeeee
 s

241
VFVFFDLTVD
bbbbeebeee

251
DQSVYPKALR
eeeeeeeebe

261
DEYIMSKTLG
eebebbeeee
  s     ff

271
SGACGEVKLA
eeebbebebb
 ff s s

281
FERKTCKKVA
beeeebeebb
         s

291
IKIISKRKFA
beebeeeeee
 f   f

301
IGSAREADPA
eeeeeeeeee

311
LNVETEIEIL
eebeeebebb
     f

321
KKLNHPCIIK
eebeeebbbe
    ff

331
IKNFFDAEDY
beebbeeeeb

341
YIVLELMEGG
bbbbebbeee
   sf   ff

351
ELFDKVVGNK
ebbeebeeee
fs

361
RLKEATCKLY
ebeeeebeeb
   f   f

371
FYQMLLAVQY
bbebbebbeb
  f      s

381
LHENGIIHRD
beeeebbbee
sf   s sff

391
LKPENVLLSS
beeeebbbee
sfff   s

401
QEEDCLIKIT
eeeebbbebb
       f

411
DFGHSKILGE
ebebbebbee
fsf sf

421
TSLMRTLCGT
eebbeebbee
     f sff

431
PTYLAPEVLV
eeebbeebbe
f f sff

441
SVGTAGYNRA
eeeeeeebee
      f

451
VDCWSLGVIL
bebbbbbbbb
sf ss ss

461
FICLSGYPPF
bbbbbbeeee
   s s  ff

471
SEHRTQVSLK
eeeeeeeebe

481
DQITSGKYNF
eebeebebeb
 fs

491
IPEVWAEVSE
eeeebeebee
    s   f

501
KALDLVKKLL
ebeebbeebb
 s

511
VVDPKARFTT
ebeeeeebeb
 s    f

521
EEALRHPWLQ
eebbeeebbe
     f s

531
DEDMKRKFQD
eeebeeebee

541
LLSEENESTA
bbeeeeeeee

551
LPQVLAQPST
eeeeeeeeee

561
SRKRPREGEA
eeeeeeeeee

571
EGAETTKRPA
eeeeeeeeeb

581
VCAAVL
bbeeee
```

The conservation scale:

1	2	3	4	5	6	7	8	9

Variable **Average** **Conservation**

e - An exposed residue according to the neural-network algorithm.

b - A buried residue according to the neural-network algorithm.

f - A predicted functional residue (highly conserved and exposed).

s - A predicted structural residue (highly conserved and buried).

X - Insufficient data - the calculation for this site was performed on less than 10% of the sequences.
